# A novel genotype of *Hepacivirus bovis* identified in reindeer (*Rangifer tarandus*) in northeastern China

**DOI:** 10.3389/fcimb.2025.1646191

**Published:** 2025-09-08

**Authors:** Wenbo Xu, Wei Wang, Liyan Sui, Nan Liu, Yinghua Zhao, Quan Liu

**Affiliations:** ^1^ Department of Infectious Diseases and Center for Infectious Diseases and Pathogen Biology, Key Laboratory of Organ Regeneration and Transplantation of the Ministry of Education, State Key Laboratory for Diagnosis and Treatment of Severe Zoonotic Infectious Diseases, Key Laboratory for Zoonosis Research of the Ministry of Education, The First Hospital of Jilin University, Changchun, China; ^2^ Hulunbuir Animal Disease Control Center, Hailar, China; ^3^ Guangdong Key Laboratory of Animal Conservation and Resource Utilization, Institute of Zoology, Guangdong Academy of Sciences, Guangzhou, Guangdong, China

**Keywords:** hepacivirus, *Rangifer tarandus*, metagenomic sequence, co-evolution, Northeastern China

## Abstract

**Background:**

Hepaciviruses (family *Flaviviridae*) are significant pathogens affecting both human and animal health. While the hepatitis C virus (*Hepacivirus hominis*) is extensively studies in humans, related viruses have been identified across various animal species. Bovine hepacivirus (BovHepV) is capable of persistent infection in cattle, facilitating mutation accumulation and recombination events that may generate novel variants. BovHepV has also been found in wild boars and sheep, suggesting a broader host range than previously recognized.

**Methods:**

In this study, metagenomic sequencing was performed on 21 serum samples collected from reindeer (*Rangifer tarandus*) in Inner Mongolia, China. Two near-complete hepacivirus genomes were identified and designated as *Rangifer tarandus* hepacivirus (RtHepV) isolates GH01 and GH02. Phylogenetic and *p*-distance analyses were used to assess genetic relatedness to known hepaciviruses. Recombination detection and host–virus co-evolutionary analyses were also conducted.

**Results:**

Among 21 reindeer serum samples, the positivity rates of RtHepV GH01 and GH02 were 42.9% (9/21) and 4.8% (1/21), respectively. These isolates shared the highest sequence identities with the BovHepV Bulgaria 9 strain, with nucleotide identities of 68.2% (GH01) and 67.9% (GH02), and amino acid identities of 75.0% (GH01) and 74.8% (GH02). Phylogenetic analysis clustered RtHepV within the *Hepacivirus bovis* lineage, but in a distinct clade separate from previously reported BovHepV strains. *P*-distance calculations indicated that RtHepV does not constitute a novel species; instead, it qualifies as a novel genotype within *Hepacivirus bovis*, as its amino acid identity with other subtypes falls below the 77% threshold. Recombination analyses revealed evidence of genetic exchange between RtHepV and BovHepV strains. Co-evolutionary analyses further highlighted frequent host-switching events within the genus *Hepacivirus*.

**Conclusion:**

This study reports the identification of two novel hepacivirus variants in reindeer from northeastern China, closely related to bovine hepaciviruses. These findings expand the known host range and geographic distribution of Hepacivirus, highlighting its ecological adaptability and the risk of cross-species transmission. The results underscore the potential public and veterinary health implications of hepaciviruses, warranting further investigation into the epidemiology of hepaciviruses.

## 1 Introduction

Hepacivirus, a member of the family *Flaviviridae*, is an enveloped virus with a positive-sense, single-stranded RNA genome. The genome spans approximately 8.9–10.5 kb and contains a single open reading frame (ORF) flanked by 5’ and 3’ untranslated regions. The ORF encodes a polyprotein that is processed by both viral and host proteases into ten distinct proteins: three structural proteins (core, E1, and E2) and seven non-structural proteins (p7, NS2, NS3, NS4A, NS4B, NS5A, and NS5B) (Parola, 2004). Hepatitis C virus (HCV), or *Hepacivirus hominis*, is a major human pathogen responsible for cirrhosis and hepatocellular carcinoma. According to the World Health Organization, approximately 58 million people globally are living with chronic HCV infection, with around 1.5 million new cases each year. HCV-related diseases caused an estimated 290,000 deaths in 2019 (https://www.who.int/news-room/fact-sheets/detail/hepatitis-c). HCV, first identified in 1989, was long thought to infect only humans as its natural host (Alter, 1989). However, this notion was challenged in 2011 with the identification of a novel HCV homolog in the respiratory tract of dogs (Kapoor et al., 2011). Subsequent studies have identified a diverse range of hepaciviruses in various hosts, including mammals such as horses (Bezerra et al., 2022), cattle (Yeşilbağ et al., 2018), non-human primates (Simons et al., 1995), bats (Wang et al., 2017), and rodents (An et al., 2022), as well as non-mammalian species like ducks (Zhang et al., 2022). These hepaciviruses have been classified into at least 14 distinct species, with recent additions such as *Hepacivirus P* (Li et al., 2019) and *Hepacivirus Q* (Zhang et al., 2022). Furthermore, advances in metagenomic sequencing have enabled the identification of hepaciviruses and hepacivirus-like viruses in diverse hosts, including marine organisms (e.g., *Proscyllium habereri*, *Mauremys megalocephala*, *Rhinobatos hynnicephalus*) (Shi et al., 2016, 2018), terrestrial reptiles (*Teratoscincus roborowskii*) (Shi et al., 2018), birds (*Cyanistes caeruleus*) (Porter et al., 2020), and invertebrates like ticks (Shao et al., 2021) and mosquitoes (Williams et al., 2020). These findings highlight the remarkable genetic diversity and broad host range of these viruses.

Currently, bovine hepacivirus (BovHepV) is recognized as the sole member of the species *Hepacivirus bovis* (formerly known as *Hepacivirus N*). It is classified into two genotypes, with genotype 1 further divided into eight subtypes (A–H). Recent studies in Bulgaria have identified novel BovHepV variants, provisionally designated as subtypes I to K (Breitfeld et al., 2022). Notably, several host spillover events have been reported, with BovHepV detected in red deer (Breitfeld et al., 2022), sheep (Ma et al., 2025), and wild boar (de Martinis et al., 2022), indicating that the virus lacks strict host specificity. Similar findings have been reported for equine hepacivirus (EqHV), which has been detected in donkeys and dogs (Pybus and Thézé, 2016; Walter et al., 2017). These studies suggest the potential for interspecies transmission of BovHepV, but knowledge of its transmission routes and host range remains limited.

Reindeer (*Rangifer tarandus*) are the only fully domesticated species within the *Cervidae* family. They inhabit tundra, Arctic, and subarctic regions across Asia, the Americas, and Europe (Li et al., 2017). In China, the Ewenki people of the northern Greater Khingan Mountains practice reindeer herding, a tradition of great cultural and economic importance (Zhai et al., 2017). However, the frequent daily interactions between herders and reindeer increase the potential risk of cross-species transmission of zoonotic pathogens. Reindeer have been recognized as sentinel species for monitoring a wide range of viruses, including multiple zoonotic pathogens (Paulsen et al., 2020; Lamsal et al., 2023). These viruses sustain a natural transmission cycle within a region, involving humans, ruminants, and arthropods (Sánchez Romano et al., 2019). A study demonstrated the transmission of hepatitis E virus between reindeer and their herders, suggesting that both may serve as natural hosts for various zoonotic viruses (Slukinova et al., 2021). Moreover, with the growth of reindeer-themed tourism in China, activities such as feeding, petting, and other forms of close interaction between tourists and reindeer have become more prevalent. This trend has not only expanded the number of individuals exposed to reindeer but also broadened the scope and frequency of such interactions.

Moreover, with the growth of reindeer-themed tourism in China, activities such as feeding, petting, and other forms of close interaction between tourists and reindeer have become more prevalent. This trend has not only expanded the number of individuals exposed to reindeer but also broadened the scope and frequency of such interactions.

This study identified a new genotype of *Hepacivirus bovis* in reindeer from Northeastern China, expanding our understanding of the diversity and distribution of this viral genus. *Hepacivirus bovis* has been detected in cattle, wild boars, and sheep (Ma et al., 2025), and the present study reports, for the first time, its presence in reindeer, further expanding the known host range of the virus. These findings suggest that reindeer may serve as a novel natural host, contributing to the ecological persistence and transmission cycle of *Hepacivirus bovis*, and offers valuable insights for enhancing surveillance strategies targeting zoonotic viruses. The ongoing discovery of hepacivirus variants across various hosts underscores the necessity for continuous surveillance and research to fully understand their impact on animal and human health.

## 2 Materials and methods

### 2.1 Sample collection and ethics

In June 2022, 21 blood samples were collected from reindeer in Inner Mongolia Autonomous Region, China, via jugular vein puncture by professional veterinarians to minimize tissue damage. The samples were centrifuged at 3,000 rpm for 10 minutes to separate serum, which was then stored at -80°C for subsequent analysis. This study was approved by the Animal Management and Ethics Committee of the First Hospital of Jilin University, and all procedures strictly complied with the Ethical Principles and Guidelines for Animal Experimentation in the People’s Republic of China.

### 2.2 RNA extraction and metagenome sequencing analysis

From each sample, 50 μL of serum was collected and combined into a pooled sample. The pooled samples were digested with micrococcal nuclease (NEB, USA) at 37°C for 2 hours. Total viral RNA was then extracted from the digested samples using the TIANamp Virus RNA kit (TIANGEN, China) according to the manufacturer’s instructions for metagenomic sequencing. The metagenomic sequencing procedure, described previously [27], involved fragmenting the RNA and reverse-transcribing it into cDNA. The cDNA fragments underwent end repair, followed by ligation with sequencing adapters using the TruSeq™ DNA Sample Prep Kit (Illumina) to construct sequencing libraries. Bridge PCR was subsequently performed to amplify adapter-ligated DNA fragments on the sequencing flow cell. Sequencing was carried out on the Illumina NovaSeq 6000 platform. After filtering out low-quality reads and adapter sequences, the raw data were further processed using BBMap (https://github.com/BioInfoTools/bbmap) to remove host contamination and rRNA, yielding clean data. *De novo* assembly of clean reads was conducted using SPAdes (https://github.com/ablab/spades) and SOAPdenovo (https://github.com/aquaskyline/SOAPdenovo-Trans). The assembled contigs were compared against the virus-NT database using BLAST (V2.10.0+) to identify viral species and infer evolutionary relationships.

### 2.3 Polymerase chain reaction

To validate the accuracy of the assembled hepacivirus sequences, primers spanning the entire viral genome were designed (**Supplementary Table S1**). RNA was extracted from each individual sample and subjected to reverse transcription followed by nested PCR. The PCR reaction mixture contained 12.5 μL of Premix Taq (TaKaRa), 9.5 μL of ddH_2_O, 1 μL each of forward and reverse primers, and 1 μL of cDNA. Thermal cycling conditions were as follows: initial denaturation at 94°C for 5 minutes; 35 cycles of denaturation at 94°C for 30 seconds, annealing at 50°C for 30 seconds, and extension at 72°C for 30 seconds; and a final extension at 72°C for 5 minutes. For the second round of PCR, the products from the first round were used as templates under the same conditions. The amplified PCR products were subsequently subjected to Sanger sequencing.

### 2.4 Rapid-amplification of cDNA ends

The 5′ and 3′ ends of the viral genome were amplified using the SMARTer^®^ RACE 5’/3’ Kit (TaKaRa) (Lu et al., 2025). RACE-PCR was performed with universal and gene-specific primers (**Supplementary Table S1**). PCR products were cloned into the pMD19-T vector (TaKaRa) and transformed into Stellar competent cells. Recombinant clones were sequenced by Sanger sequencing at Sangon Biotech (Shanghai) Co., Ltd., and the data were used to assemble the complete viral genome.

### 2.5 Genome annotation and phylogenetic analysis

The assembled viral sequences were analyzed using online BLASTn searches against the Nr/Nt database (https://blast.ncbi.nlm.nih.gov/Blast.cgi). ORFs were predicted using ORFfinder (https://www.ncbi.nlm.nih.gov/orffinder). Cleavage sites in viral proteins were inferred by comparing the protein sequences with those of BovHepV Bulgaria 9 strain (ON402465). N-glycosylation sites in the E1 and E2 proteins were predicted via the NetNGlyc 1.0 service (https://services.healthtech.dtu.dk/service.php?NetNGlyc-1.0). All hepacivirus sequences were retrieved from the GenBank database and aligned with ClustalW (**Supplementary Table S2**). Nucleotide (nt) and amino acid (aa) sequence identities were calculated using MegAlign in DNAstar (v7.1). Average amino acid p-distances between sequence groups were calculated using the Simple Sequences Editor (SSE) v1.4, generating amino acid distance line graphs (window size: 200 residues, step size: 20) (Smith et al., 2016). Evolutionary relationships were inferred by maximum likelihood (ML) analysis using MEGA v7.0. Bootstrap analysis was performed with 1,000 replicates; values above 70 were considered significant and are shown on the phylogenetic tree.

### 2.6 Recombination analysis

The RDP4 package was employed to detect potential recombination events in Hepacivirus bovis (Martin et al., 2015). Recombination analysis was conducted on aligned sequences using seven detection methods (RDP, GENECONV, BootScan, MaxChi, Chimaera, SiScan, and 3Seq) under default parameters. A recombination event was considered valid only if it was detected by at least two independent methods and met the Bonferroni-corrected significance threshold of *p* < 0.007 (0.05/7). Additionally, RDP-identified recombination events with RDP recombination confidence scores ranging from 0.40 to 0.60 were classified as potential recombination events (Ma et al., 2025).

### 2.7 Co-evolution analyses

To investigate the co-evolution between hepaciviruses and their vertebrate hosts, a host species evolutionary tree was constructed using TimeTree 5 (http://www.timetree.org). Hepacivirus phylogenetic events were mapped onto the host tree using Jane 4 (Conow et al., 2010). The mapping sought to minimize total cost according to set values: 0 for cospeciation, and 1 each for duplication, host switch, loss, and failure to diverge (Shi et al., 2018). TreeMap3 (http://sites.google.com/site/cophylogeny) was used to visualize host-virus associations. The untangle function minimized crossing lines, and default settings estimated the relative frequency of co-evolution events.

## 3 Result

### 3.1 Virus identification and genomic characterization

In June 2022, blood samples from 21 reindeer were collected to construct an RNA library for metagenomic sequencing. The Metagenomic sequencing yielded 6.4 gigabytes of data, from which 38.5 million high-quality reads were obtained after filtering out low-quality and host-derived sequences. Eleven contigs related to BovHepV were identified in the dataset. Using RACE, two nearly complete viral genomes were amplified and designated as *Rangifer tarandus* hepacivirus (RtHepV) GH01 and GH02 (accession numbers OQ164634–OQ164635). All 21 samples were tested for RtHepV, with a positivity rate of 42.9% (9/21) for GH01 and 4.8% (1/21) for GH02. No co-infection with both GH01 and GH02 strains was detected.

**Figure 1:**
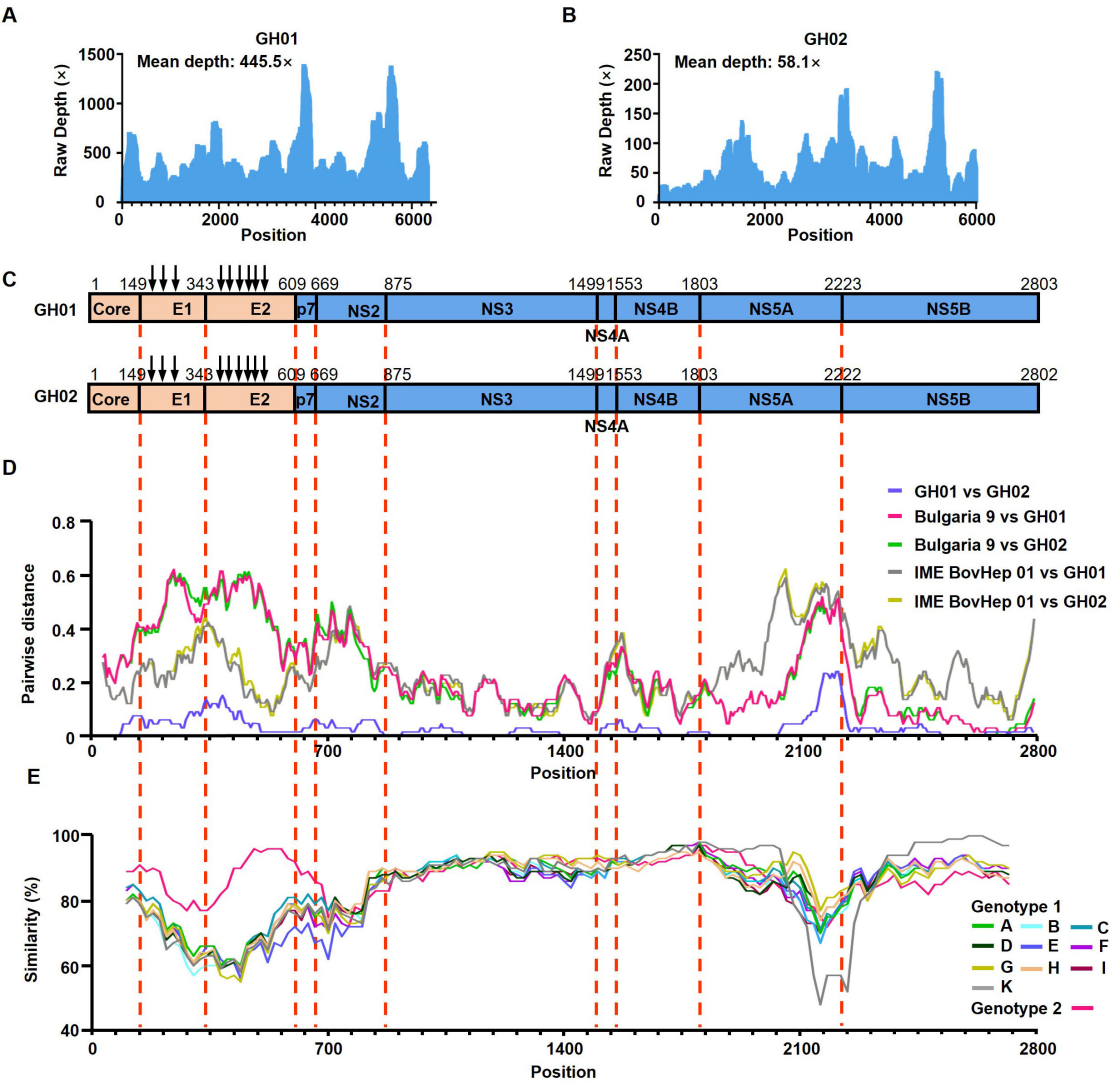
Genomic characteristics of *Rangifer tarandus* hepacivirus (RtHepV). **(A, B)** Histograms showing the sequencing depth of mapped reads, illustrating the distribution and abundance of sequencing reads across the RtHepV genome. **(C)** Schematic representation of the genome structure and predicted polyprotein cleavage sites, based on alignment with the Bovine hepacivirus strain BovHepV Bulgaria 9 (ON402465). Vertical arrows indicate predicted N-linked glycosylation sites. **(D)** Amino acid p-distance analysis of RtHepV compared with Bovine hepacivirus isolates BovHepV Bulgaria 9 and IME BovHep 01(MN691105), calculated using SSE v1.4 (window size = 200, step size = 20). **(E)** Amino acid identity analysis among different *Hepacivirus bovis* genotypes, performed using Simplot v3.5.1 with a sliding window of 200 and a step size of 20.

The average sequencing depths for the GH01 and GH02 strains were 445.5 and 58.1, respectively (**Figures 1A, B**). Both strains exhibited similar GC content: 53.6% for GH01 and 53.4% for GH02. The GH01 genome consisted of 8,904 nucleotides and encoded a polyprotein of 2,802 amino acids. Compared to GH01, GH02 lacked three nucleotides at positions 6860–6862, leading to the deletion of one amino acid in the NS5A protein (**Figure 1C**). Genome annotation revealed highly conserved cleavage sites within the polyproteins of both viral strains. Additionally, three N-glycosylation sites were predicted in the E1 protein and six in the E2 protein (**Figure 1C**).

### 3.2 Sequence comparison

The two RtHepV strains, GH01 and GH02, share a high nucleotide identity of 90.2% and an amino acid identity of 96.8%. Both strains exhibit the highest sequence similarity with the Bovine hepacivirus isolate BovHepV Bulgaria 9 (accession no. ON402465), with nucleotide identities of 68.2% (GH01) and 67.9% (GH02), and amino acid identities of 75.0% (GH01) and 74.8% (GH02). Compared to other BovHepV subtypes, their nucleotide identity ranges from 62.4% to 65.5%, and amino acid identity from 69.5% to 74.7%. In contrast, their similarity to other hepacivirus species is notably lower, with nucleotide identities ranging from 22.2% to 32.9%, and amino acid identities from 33.8% to 41.4% (**Table 1**).

**Table 1:** Nucleotide (upper right) and amino acid (lower left) sequence identity analysis of hepacivirus polyprotein.

Strains	1	2	3	4	5	6	7	8	9	10	11	12	13	14	15	16	17	18	19	20	21	22	23	24	25	26	27	28	29	30	31	32
1. OQ164635 GH02/China		90.2	36.1	36.1	40.1	34.0	34.8	35.2	39.0	38.6	37.6	37.8	39.3	36.6	34.8	41.4	35.5	68.2	62.8	62.5	62.4	62.7	62.8	63.2	62.8	62.8	63.0	62.7	63.3	65.5	33.8	39.9
2. OQ164634 GH01/China	96.8		36.6	36.6	39.9	34.1	34.6	35.3	39.1	38.5	37.9	38.1	39.2	36.6	35.0	41.0	35.7	67.9	62.6	62.6	62.7	63.1	63.0	63.0	62.7	62.8	62.9	62.6	63.3	65.4	34.1	40.2
3. JF744991 AAK-2011/USA	26.1	26.0		99.6	34.2	48.2	48.4	31.9	34.3	33.4	33.7	34.3	35.0	34.8	42.8	35.2	42.8	36.0	36.2	36.4	36.3	36.2	36.2	35.9	36.1	36.1	36.7	36.4	36.7	36.2	33.3	35.0
4. KP325401 NZP1	26.1	25.9	99.6		34.2	48.2	48.4	31.9	34.3	33.5	33.6	34.4	35.0	34.8	42.7	35.2	42.7	36.0	36.1	36.3	36.1	36.2	36.1	35.8	36.0	36.1	36.7	36.3	36.6	36.1	33.3	34.9
5. U22304 Hepacivirus B/USA	31.4	31.4	24.4	24.3		33.4	33.2	36.4	38.6	38.2	37.7	38.0	39.1	35.8	34.4	43.3	34.6	40.4	40.6	39.9	40.2	40.0	40.3	40.3	40.2	40.8	40.4	39.9	40.5	40.3	33.2	39.3
6. NC009826 EUH1480/UK	24.2	24.2	44.9	44.9	23.7		67.4	31.0	33.8	33.9	33.6	34.6	33.7	35.9	42.7	35.1	41.4	33.4	33.8	34.5	33.7	33.4	33.5	33.7	33.8	33.8	34.0	33.8	33.8	34.2	32.0	34.0
7. NC009827 Th580	24.4	24.5	45.6	45.6	23.6	74.3		30.9	33.8	33.5	33.2	34.1	34.3	35.4	42.9	34.7	41.0	33.9	34.4	34.5	34.0	34.1	34.5	34.0	34.3	34.6	34.0	34.3	34.6	34.0	32.2	34.2
8. KC551800 GHV-1 BWC08/Uganda	27.8	27.9	22.1	22.0	29.3	21.2	21.4		34.3	34.7	34.1	35.2	35.0	34.2	32.3	39.6	31.6	35.6	35.2	35.4	35.5	35.4	35.2	35.4	35.3	35.6	35.1	35.4	35.6	34.9	31.8	34.5
9. KC815310 RHV-339/USA	30.9	30.9	24.1	24.2	31.7	23.1	23.4	26.7		57.1	48.8	47.5	40.0	35.5	34.7	40.2	35.0	39.1	39.3	39.1	39.3	39.4	39.5	39.2	39.2	39.0	39.1	38.9	39.3	39.7	32.5	50.5
10.KC411784 Hepacivirus/NLR07-oct70/NEL/2007/Netherlands	30.0	29.9	23.9	24.0	31.3	23.2	22.9	27.4	60.7		50.0	46.8	38.8	36.8	35.4	39.1	35.1	38.5	37.6	37.9	37.8	38.4	37.7	38.5	37.8	38.0	38.2	37.6	38.1	38.4	32.5	49.8
11. KJ950938 NrHV-1/NYC-C12/USA	29.0	28.9	23.8	23.8	31.3	23.0	23.1	26.1	48.0	47.3		46.6	37.8	36.2	35.6	38.9	35.1	37.2	37.7	37.2	37.3	37.3	37.0	37.1	37.5	37.2	37.6	37.1	37.1	37.4	31.7	47.5
12. KJ950939 NrHV-2/NYC-E43/USA	29.1	29.1	23.9	23.9	30.4	23.7	24.0	26.9	42.8	41.3	41.3		39.8	35.5	35.4	39.6	34.7	38.1	38.2	37.7	38.3	38.2	38.1	38.1	37.8	38.1	38.1	38.1	38.0	39.2	31.8	48.7
13. KC411806 Hepacivirus/SAR-3/RSA/2008/South Africa	29.9	30.1	24.3	24.3	30.3	23.7	24.7	27.0	31.1	29.9	29.8	30.7		36.2	35.5	40.5	35.6	40.0	39.2	39.5	39.7	39.3	40.2	39.7	39.7	40.1	39.8	39.5	40.1	39.2	32.5	40.9
14. KC411777 Hepacivirus/RMU10-3382/GER/2010/Germany	25.9	25.9	24.5	24.4	26.0	24.2	25.0	23.5	25.0	25.3	24.8	24.0	25.7		37.7	36.3	36.9	36.9	36.9	36.8	36.6	36.5	36.8	37.2	36.3	37.0	36.8	36.8	36.7	36.4	34.9	35.3
15. KC796074 PDB-829/Kenya	25.3	25.2	34.4	34.3	24.7	33.3	33.8	22.5	24.7	24.6	24.3	25.2	26.3	25.9		35.8	51.4	34.9	35.1	35.3	34.8	34.8	35.0	35.3	35.3	35.0	35.0	35.3	35.2	35.5	33.6	35.1
16. KC796077 PDB-112/Kenya	32.9	32.8	25.8	25.8	36.6	24.7	24.8	33.7	32.6	32.0	31.6	31.0	31.7	26.7	25.3		34.9	40.7	40.4	40.8	40.4	40.3	40.7	40.8	41.1	40.7	40.5	40.4	40.7	41.4	33.5	40.5
17. KC796078 PDB-491.1/Kenya	25.9	25.9	35.7	35.6	24.6	33.3	33.2	22.5	25.4	24.9	25.0	25.5	26.3	26.0	48.4	25.2		35.4	35.2	35.1	35.2	34.8	35.3	35.8	35.1	35.0	35.1	35.0	35.5	35.4	33.9	35.0
18. ON402465 BovHepV Bulgaria 9/Bulgaria	75.0	74.8	25.7	25.6	31.7	23.4	23.9	27.3	30.5	30.0	29.3	29.3	30.4	26.8	25.2	32.7	25.6		72.6	73.1	72.2	72.5	73.4	73.8	72.7	73.0	72.9	72.2	74.4	63.8	34.4	40.3
19. MZ221927 GDZJ/China	70.0	69.7	25.6	25.6	31.8	23.5	23.6	27.0	30.4	29.6	29.0	29.5	30.0	26.6	25.3	31.5	25.2	82.8		84.4	81.6	81.6	80.0	79.6	80.5	79.5	80.2	81.5	80.2	66.4	34.1	39.9
20. MG781018 BR MA236B017/Brazil	70.2	69.9	25.6	25.6	31.5	23.3	23.7	26.8	30.1	29.4	28.9	29.3	29.9	26.5	25.2	31.8	25.3	83.4	95.6		82.1	82.4	80.7	80.8	80.1	79.9	80.5	82.1	81.1	66.4	34.4	39.9
21. MW830376 CQ/166/China	69.7	69.8	25.2	25.2	31.5	23.1	23.1	26.8	30.3	29.3	28.7	29.4	30.1	26.5	25.1	31.8	25.2	82.7	94.2	94.3		84.4	80.1	80.3	79.1	79.4	79.8	92.4	80.2	67.0	33.7	40.1
22. KP641125 BovHepV 379/Ger/2014/Germany	70.4	70.3	25.2	25.2	31.4	23.3	23.2	26.8	30.4	29.7	28.8	29.3	29.7	26.7	25.3	31.7	25.2	83.3	94.3	94.4	95.8		80.1	80.2	79.4	79.9	79.6	84.6	80.6	66.3	33.9	39.8
23. KP265950 GHC100/Ghana	70.3	69.9	25.7	25.6	31.6	23.4	23.5	26.9	30.3	29.5	29.0	29.1	30.1	26.8	25.2	32.2	25.2	84.0	92.5	93.2	92.6	93.0		82.5	81.9	81.4	81.4	79.8	82.5	66.8	33.5	40.1
24. KP265946 GHC52/Ghana	70.6	70.4	25.5	25.5	31.7	23.4	23.6	26.8	30.6	29.5	29.0	29.3	30.1	26.8	25.3	32.2	25.4	83.5	91.9	92.5	92.0	92.2	95.4		81.3	81.2	81.9	80.0	86.7	66.5	33.9	39.9
25. MG257793 BovHepV/GD/01/China	69.9	70.0	25.6	25.6	31.5	23.1	23.2	26.7	30.3	29.4	29.1	29.4	30.2	26.4	25.3	32.0	25.2	83.3	91.7	92.4	91.9	91.9	95.3	94.3		80.9	81.4	79.1	81.6	66.6	33.7	39.9
26. MH027948 BH181/16-20/Germany	70.1	69.9	25.8	25.8	31.8	23.4	23.6	26.9	30.6	29.7	29.1	29.3	30.3	27.0	25.4	32.2	25.5	83.6	91.9	92.4	91.7	92.0	95.1	94.3	94.2		82.1	79.2	81.5	67.0	33.8	40.0
27. ON402464 BovHepV Bulgaria 19/Bulgaria	70.1	70.0	25.8	25.8	31.7	23.3	23.4	26.7	30.6	29.7	29.1	29.5	30.3	26.7	25.3	32.1	25.3	83.4	92.4	92.4	92.3	92.9	95.5	94.5	94.3	95.5		79.5	81.8	66.7	33.9	40.0
28. OP716809 HLJ-72/China	69.6	69.5	25.5	25.4	31.6	23.2	23.2	27.0	30.0	29.1	28.7	29.2	29.9	26.5	25.2	31.6	25.2	82.6	94.2	94.5	97.2	95.5	92.5	91.9	91.7	91.6	92.2		80.1	66.8	33.8	40.1
29. OU592967 Bovine hepacivirus	70.3	70.1	25.8	25.7	31.7	23.3	23.6	26.8	30.6	29.5	29.2	29.3	30.1	26.7	25.3	32.0	25.4	83.5	91.6	92.5	91.9	92.1	95.3	96.3	94.3	94.2	94.5	91.9		66.7	33.5	39.8
30. MN691105 IME BovHep 01/China	74.6	74.7	25.4	25.3	31.6	23.4	23.6	26.7	30.8	29.5	29.1	29.9	30.4	26.4	25.4	32.5	25.4	72.6	76.3	76.5	76.5	76.3	76.0	76.3	75.8	76.3	76.6	76.3	75.9		33.5	39.7
31. MG211815 RHV-GS2015/China	31.7	31.7	24.8	24.9	32.3	23.8	24.6	27.1	48.9	47.8	45.0	44.2	33.2	25.8	25.7	33.8	25.9	31.9	31.3	31.1	31.2	31.3	31.2	31.2	30.9	31.3	31.4	31.2	31.2	31.5		33.2
32. OM203121 GDZQ-15/China	22.2	22.3	20.5	20.5	21.3	20.1	20.4	19.9	21.2	20.8	20.9	20.5	21.0	22.2	21.7	22.3	22.2	21.8	21.6	21.4	21.6	21.7	21.7	21.6	21.7	21.9	21.7	21.6	21.5	21.7	21.6	

Based on *p*-distance analysis of the conserved NS3 and NS5B regions, GH01 and GH02 do not meet the species demarcation threshold for classification as a novel *Hepacivirus* species (**Figure 1D**). However, their amino acid identities with other BovHepV isolates fall below 77%, supporting their classification as a novel genotype. Thus, they are provisionally assigned as genotype 3 (**Table 1**).

### 3.3 Phylogenetic analysis and homologous recombination

**Figure 2:**
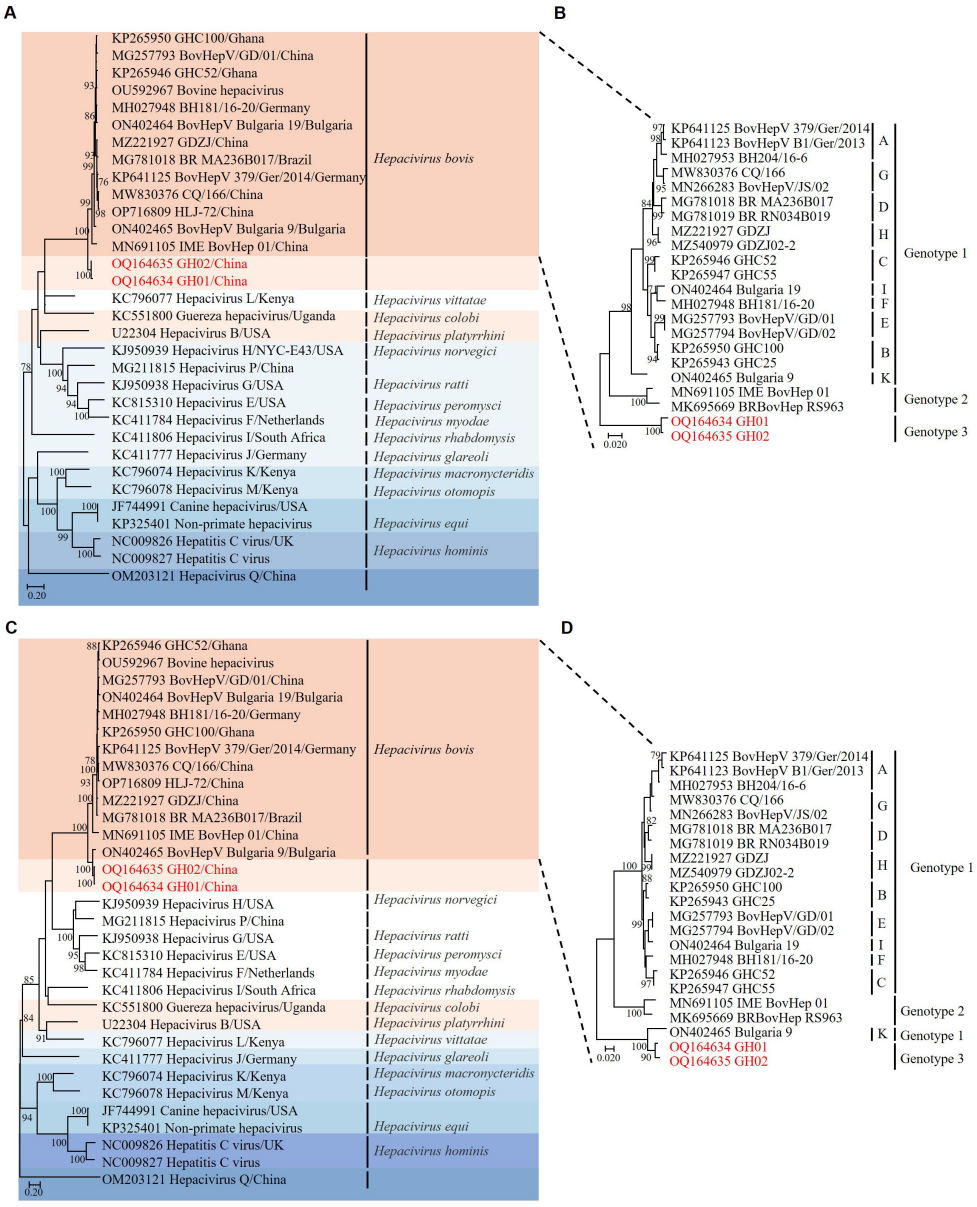
Phylogenetic trees based on amino acid sequences of hepaciviruses. **(A)** Phylogenetic analysis of hepaciviruses based on NS3 amino acid sequences. **(B)** Phylogenetic analysis of *Hepacivirus bovis* based on NS3 amino acid sequences. **(C)** Phylogenetic analysis of hepaciviruses based on NS5B amino acid sequences. **(D)** Phylogenetic analysis of *Hepacivirus bovis* based on NS5B amino acid sequences. The J subtype was excluded due to the absence of a complete genome sequence. Bootstrap values greater than 70 were considered significant and are indicated on the trees. The *Rangifer tarandus* hepaciviruses identified in this study are highlighted in red.

Phylogenetic analysis based on the amino acid sequences of the NS3 region revealed that RtHepV clustered within *Hepacivirus bovis*, forming a distinct branch closely related to Bovine hepacivirus (BovHepV) (**Figures 2A, B**). However, a topological shift was observed in the NS5B-based phylogenetic tree: the BovHepV Bulgaria 9 strain, which was previously classified as genotype 1, clustered within genotype 3 together with RtHepV (**Figures 2C, D**).

Homology analysis of individual viral proteins demonstrated a high similarity between the GH01 and GH02 strains of RtHepV, with nucleotide identities ranging from 87.0% to 93.3% and amino acid identities from 94.2% to 98.8%. Among BovHepV strains, the core-to-p7 region of GH01 exhibited the greatest similarity to the IME BovHep 01 strain, with nucleotide identities ranging from 62.0% to 70.5% and amino acid identities between 72.1% and 83.3%. In the NS2–NS3 region, GH01 shared amino acid identities of 63.5% in NS2 and 85.0% in NS3 with both IME BovHep 01 and BovHepV Bulgaria 9. Within the NS4A–NS5B region, GH01 showed the highest similarity to BovHepV Bulgaria 9, with nucleotide identities ranging from 66.0% to 81.6% and amino acid identities from 67.0% to 93.1% (**Table 2**).

**Table 2:** Nucleotide (italicized) and amino acid (regular) sequence identity analysis of individual proteins of Hepacivirus bovis*.

Acc. Nos.	Genotype	Subtype	Strain	Sequence identity (%)									
				Core	E1	E2	p7	NS2	NS3	NS4A	NS4B	NS5A	NS5B
OQ164635	3	—	GH02	92.6	90.5	90.1	89.4	88.6	89.2	87.0	90.4	88.0	93.3
				*96.6*	*94.3*	*94.3*	*96.6*	*96.1*	*98.8*	*94.4*	*98.4*	*94.2*	*98.4*
KP641125	1	A	BovHepV 379/Ger/2014	53.9	56.5	53.9	66.6	58.5	71.2	64.8	71.8	50.7	68.1
				*63.6*	*54.1*	*49.0*	*78.3*	*61.1*	*86.5*	*74.0*	*86.0*	*53.3*	*77.7*
KP265950		B	GHC100	55.2	56.5	53.2	62.7	59.7	70.8	64.8	72.4	52.0	67.5
				*61.7*	*52.0*	*50.1*	*73.3*	*61.6*	*84.7*	*72.2*	*85.6*	*54.7*	*77.9*
KP265946		C	GHC52	54.1	57.9	54.8	62.7	57.2	72.3	62.9	71.7	53.1	67.2
				*63.0*	*52.5*	*50.9*	*75.0*	*60.1*	*85.8*	*74.0*	*84.8*	*55.4*	*78.4*
MG781018		D	BR MA236B017	54.9	55.4	53.9	65.5	57.1	70.8	61.7	72.1	51.8	67.1
				*62.4*	*51.0*	*49.0*	*76.6*	*61.1*	*85.8*	*72.2*	*85.6*	*53.5*	*77.9*
MG257793		E	BovHepV/GD/01	55.0	55.4	53.1	61.6	58.4	70.3	60.4	71.8	54.0	67.8
				*61.3*	*52.5*	*47.9*	*71.6*	*60.6*	*85.5*	*74.0*	*86.0*	*55.6*	*78.1*
MH027948		F	BH181/16-20	57.3	58.9	53.0	65.0	58.4	69.6	62.3	72.6	53.6	67.0
				*63.0*	*52.0*	*49.4*	*73.3*	*60.6*	*84.4*	*72.2*	*86.8*	*54.9*	*78.1*
MW830376		G	CQ/166	54.3	56.7	53.6	67.2	57.4	70.2	61.1	71.2	51.3	67.8
				*62.4*	*52.0*	*49.0*	*78.3*	*57.2*	*86.3*	*72.2*	*85.6*	*53.8*	*77.7*
MZ221927		H	GDZJ	55.2	56.3	53.9	67.7	58.7	71.3	60.4	72.1	51.4	67.8
				*62.4*	*51.5*	*48.3*	*78.3*	*61.1*	*86.6*	*72.2*	*83.2*	*53.3*	*77.7*
ON402464		I	BovHepV Bulgaria 19	55.4	57.2	53.9	65.0	59.2	71.7	62.9	70.4	52.4	67.6
				*61.7*	*52.5*	*49.8*	*75.0*	*61.1*	*85.4*	*74.0*	*86.0*	*54.7*	*77.5*
ON402465		K	BovHepV Bulgaria 9	55.8	57.9	54.3	69.4	59.7	71.6	66.0	71.2	65.6	81.6
				*63.6*	*52.0*	*49.4*	*76.6*	*63.5*	*85.0*	*72.2*	*82.8*	*67.0*	*93.1*
MN691105	2	—	IME BovHep 01	62.0	68.5	69.5	70.5	60.5	71.6	60.4	69.8	54.1	65.2
				*78.1*	*72.1*	*77.5*	*83.3*	*63.5*	*85.0*	*66.6*	*83.6*	*56.4*	*75.8*

* All data are presented as homology calculations comparing different viruses with *Rangifer tarandus* hepacivirus strain GH01.

The observed patterns strongly indicated the occurrence of recombination events. Therefore, the RDP4 software package was employed to analyze homologous recombination between RtHepV and BovHepV. Seven recombination events associated with RtHepV were detected, comprising two confirmed and five potential events. In each event, RtHepV functioned as the minor parental strain, while the major parental strains were derived from BovHepV isolates reported in China (Guangdong), Germany, and Bulgaria (**Table 3**). These results imply that RtHepV not only shares evolutionary relationships with BovHepV strains in China but also exhibits distant relatedness to European strains, indicating complex evolutionary processes and possible cross-regional viral exchanges.

**Table 3:** Detection of recombination events within *Hepacivirus bovis* using RDP4 package.

Event	Recombinant	Major parent	Minor parent	Detection methods (*p*-value)							RDPRCS
				RDP	GENECONV	BootScan	MaxChi	Chimaera	Siscan	3Seq	
1	MN691105 IME BovHep 01	MZ221927 GDZJ/China	OQ164634 GH01	9.138×10^-10^	—	3.613×10^-06^	6.122×10^-04^	2.767×10^-03^	—	8.651×10^-07^	0.581
		MW830376 CQ/166	OQ164634 GH01	2.004×10^-15^	4.834×10^-06^	2.455×10^-16^	9.247×10^-03^	1.065×10^-02^	1.967×10^-35^	3.175×10^-14^	0.550
		KP641125 BovHepV 379/Ger/2014	OQ164635 GH02	1.857×10^-03^	—	—	—	1.824×10^-25^	—	—	0.525
		MG257793 BovHepV/GD/01	OQ164635 GH02	3.608×10^-02^	—	1.326×10^-02^	—	—	—	4.524×10^-02^	0.494
		Unknow	MW830376 CQ/166	—	—	—	4.989×10^-03^	—	1.107×10^-33^	—	0.475
2	ON402465 BovHepV Bulgaria 9	MH027948 BH181/16-20/Germany	OQ164635 GH02	6.247×10^-08^	2.471×10^-02^	3.037×10^-05^	4.508×10^-04^	3.202×10^-06^	2.479×10^-04^	5.650×10^-07^	0.723
		ON402464 BovHepV Bulgaria 19	OQ164634 GH01	4.321×10^-40^	4.735×10^-13^	9.771×10^-40^	8.179×10^-19^	1.277×10^-06^	2.209×10^-24^	9.525×10^-14^	0.662
		ON402464 BovHepV Bulgaria 19	OQ164635 GH02	8.599×10^-22^	—	2.950×10^-17^	5.593×10^-32^	1.066×10^-38^	2.087×10^-34^	—	0.587
		Unknow	MG257793 BovHepV/GD/01	—	—	—	2.989×10^-02^	3.484×10^-02^	—	—	0.499
3	ON402464 BovHepV Bulgaria 19/Bulgaria	MH027948 BH181/16-20/Germany	ON402465 BovHepV Bulgaria 9	—	—	—	4.978×10^-06^	2.829×10^-05^	—	—	0.629
4	KP265950 GHC100	KP265946 GHC52	Unknow	3.636×10^-02^	4.731×10^-02^	—	—	2.762×10^-02^	—	—	0.484
5	MH027948 BH181/16-20/Germany	KP265950 GHC100	MG781018 BR MA236B017/Brazil	2.555×10^-02^	—	2.692×10^-02^	—	—	—	—	0.435

### 3.4 Co-evolutionary analysis

**Figure 3:**
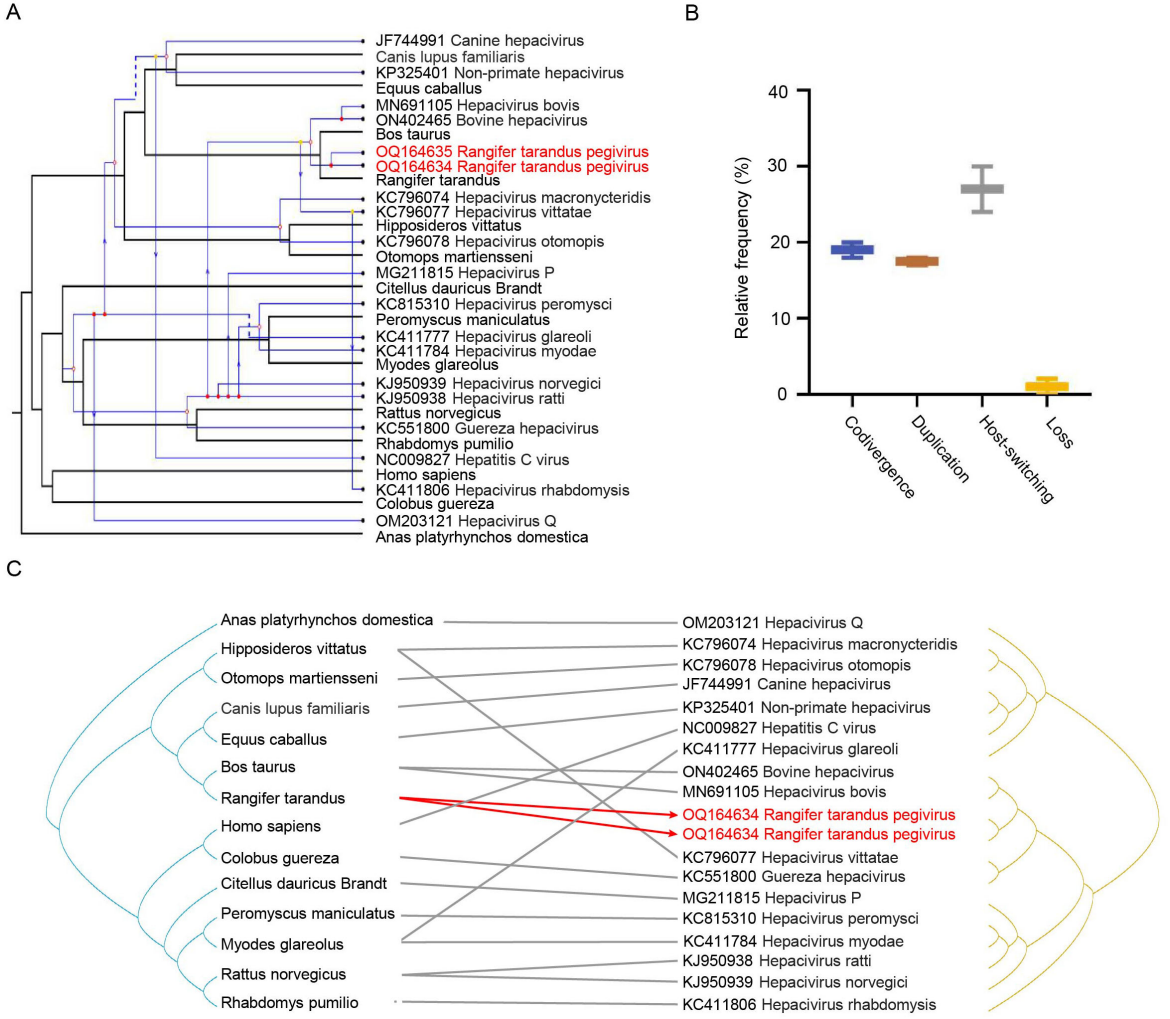
Co-evolutionary analysis of hepaciviruses. **(A)** Reconciliation of the hepacivirus phylogeny with that of their vertebrate hosts. Host and virus phylogenetic trees are shown in black and blue, respectively. Arrows indicate host-switching events; solid and hollow circles at branch tips represent co-speciation and duplication, respectively; dashed lines indicate gene loss. **(B)** Relative frequencies of four co-evolutionary events: codivergence, duplication, host-switching, and loss. **(C)** Tanglegram showing individual host–virus associations. *Rangifer tarandus* hepaciviruses identified in this study are highlighted in red.

To investigate the co-evolutionary relationships between hepaciviruses and their hosts, we utilized Jane 4 to map each evolutionary event of hepaciviruses onto the host phylogenetic tree, aiming to minimize the total reconciliation cost. The analysis identified a total of 21 evolutionary events, including 7 cospeciations, 3 duplications, 8 host-switching events, and 2 losses, with no failures to diverge (**Figure 3A**). Further analysis of the relative frequencies of four key evolutionary processes—codivergence, duplication, host-switching, and loss—revealed that host-switching was the predominant co-evolutionary mechanism, accounting for the majority (24–30 events) of occurrences (**Figure 3B**). Additionally, the tanglegram illustrating the associations between virus and host phylogenies corroborated these findings, demonstrating consistent evolutionary patterns (**Figure 3C**).

## 4 Discussion

This study identified a novel hepacivirus, designated RtHepV, in reindeer. Although it is classified within the species *Hepacivirus bovis*, RtHepV shows significant genetic divergence from all known BovHepV genotypes and has therefore been provisionally assigned to genotype 3. Unlike earlier hepacivirus spillover events, in which viruses identified in new hosts closely resembled those in the original hosts, RtHepV shares less than 77% amino acid identity with all known BovHepV subtypes. Furthermore, based on the current classification criteria of the *Hepacivirus* genus, RtHepV is the first virus within a single *hepacivirus* species to be identified from different host species. The emergence of RtHepV suggests that cross-species transmission of hepaciviruses has already occurred among distinct animal species, potentially posing significant implications for both public health and veterinary medicine.

Over the past 15 years, advances in metagenomic technologies have greatly facilitated the identification of hepaciviruses in a wide range of animal hosts. Initially thought to have narrow or host-specific ranges, these viruses have challenged previous assumptions as studies have progressed. For example, in addition to infecting humans, HCV has also been detected in non-human primates (Akari et al., 2009). Equine hepacivirus has been detected not only in donkeys but also in dogs (Pybus and Thézé, 2016; Walter et al., 2017), highlighting its potential for cross-species transmission. Zoonotic viruses are capable of crossing species barriers and are often associated with changes in virulence that can result in severe diseases, such as HIV, coronaviruses, and influenza viruse (Sharp and Hahn, 2011; Flanagan et al., 2012; Morse et al., 2012). Direct contact with animals or vector bites results in prolonged exposure to genetically diverse zoonotic viruses, increasing the risk of cross-species transmission (Mackenzie and Jeggo, 2013). Therefore, the identification and characterization of viruses from various animal origins are essential for public health safety.

The discovery of BovHepV in 2015 marked a significant milestone (Baechlein et al., 2015; Corman et al., 2015). BovHepV, the only member of *Hepacivirus bovis*, exhibits remarkable genetic diversity (Breitfeld et al., 2022). The virus has a global distribution, having been detected in seven countries across five continents (Lu et al., 2019). Current research suggests that its host range may be broader than previously thought. For instance, sequences highly homologous to BovHepV subtype F have been identified in wild boars (de Martinis et al., 2022), and BovHepV subtype G has been detected in sheep in Inner Mongolia, China, indicating a potentially wider host spectrum. Moreover, studies in red deer in the Czech Republic revealed partial NS3 coding sequences closely related to BovHepV (Breitfeld et al., 2022), suggesting that *cervids* might serve as additional reservoir hosts for *Hepacivirus bovis* extending beyond cattle.

Phylogenetic analysis revealed that RtHepV forms a distinct clade within *Hepacivirus bovis*, although topological incongruences were also observed. Although several recombination signals were detected between RtHepV and BovHepV, the methodological constraints of RDP4 warrant caution. Inferred breakpoints may fail to distinguish true recombination from convergent evolution or technical artifacts, and low RDP recombination confidence scores indicate reduced confidence. Nevertheless, these putative recombination events still provide intriguing insights into the potential origin of RtHepV. Given the well-developed livestock farming infrastructure in Inner Mongolia, reindeer and cattle share overlapping habitats, creating opportunities for cross-species viral transmission. Such transmission could occur via direct pathways (e.g., aerosolized particles or exchange of body fluids) or indirect routes (e.g., contact with contaminated feed, drinking water, feces, or carcasses). Furthermore, BovHepV has been detected in cell culture sera worldwide (Lu et al., 2019), suggesting the possibility of reindeer infection via vaccination. Mechanical transmission by vectors, especially ticks (Harvey et al., 2019; Shao et al., 2021) and tabanids (Pybus and Thézé, 2016), may facilitate contact between reindeer and cattle populations, thereby enhancing the likelihood of BovHepV transmission across species. To test this hypothesis, we collected engorged *Ixodes persulcatus* ticks from reindeer and captured free-roaming ticks of the same species from surrounding wild habitats. However, all samples tested negative for RtHepV, resulting in disappointing findings. In Inner Mongolia and other regions, reindeer and cattle engage in potential ecological interactions, including cross-regional trade and transportation. These factors may provide opportunities for genomic exchange between RtHepV and BovHepV strains from different geographic origins. In addition, the migration of wild animals may facilitate cross-regional viral dissemination. Considering these ecological and management factors, it is plausible that recombination between RtHepV and BovHepV occurs under conditions of close host contact or environmental co-exposure, highlighting the real possibility of cross-host and cross-regional genetic exchange among hepaciviruses.

Genome analysis of RtHepV revealed a single amino acid deletion in the NS5A protein of the GH02 strain, a feature with potential functional significance. NS5A is a multifunctional non-structural protein that plays critical roles in viral RNA replication, virion assembly, and modulation of host immune responses (Bulankina et al., 2022). Previous studies have shown that amino acid deletions or substitutions in NS5A can markedly affect viral replication efficiency, interfere with interferon signaling, and alter the virus’s sensitivity to host antiviral defenses (Enomoto et al., 1995; Scheel et al., 2011). Although the precise functional consequences of the GH02 NS5A deletion remain to be experimentally validated, this alteration may influence viral replication dynamics or facilitate immune evasion, potentially impacting viral fitness and pathogenicity. Future studies using reverse genetics and functional assays are warranted to elucidate the specific effects of this deletion on the biology of GH02.

In conclusion, our study underscores the increasing genetic diversity of hepaciviruses and highlights the need for sustained surveillance and research to reduce public health risks associated with cross-species transmission.

## Data Availability

The datasets presented in this study can be found in online repositories. The names of the repository/repositories and accession number(s) can be found in the article/**Supplementary Material**.

## References

[B1] Akari H. Iwasaki Y. Yoshida T. Iijima S. (2009). Non-human primate surrogate model of hepatitis C virus infection. Microbiol. Immunol. 53, 53–57. doi: 10.1111/j.1348-0421.2008.00087.x, PMID: 19161559

[B2] Alter H. J. (1989). Discovery of the non-A, non-B hepatitis virus: the end of the beginning or the beginning of the end. Transfus. Med. Rev. 3, 77–81. doi: 10.1016/s0887-7963(89)70071-7, PMID: 2562477

[B3] An C. H. Li J. Wang Y. T. Nie S. M. Chang W. H. Zhou H.. (2022). Identification of a novel hepacivirus in Mongolian gerbil (*Meriones unguiculatus*) from Shaanxi, China. Virol. Sin. 37, 307–310. doi: 10.1016/j.virs.2022.01.016, PMID: 35248515 PMC9170912

[B4] Baechlein C. Fischer N. Grundhoff A. Alawi M. Indenbirken D. Postel A.. (2015). Identification of a novel hepacivirus in domestic cattle from Germany. J. Virol. 89, 7007–7015. doi: 10.1128/JVI.00534-15, PMID: 25926652 PMC4473572

[B5] Bezerra C. S. Limeira C. H. Monteiro Dos Anjos D. Nogueira D. B. Morais D. A. Falcão B.. (2022). Global prevalence of RNA-positive horses for Hepacivirus (EqHV): Systematic review and meta-analysis. J. Equine Vet. Sci. 114, 104003. doi: 10.1016/j.jevs.2022.104003, PMID: 35508285

[B6] Breitfeld J. Fischer N. Tsachev I. Marutsov P. Baymakova M. Plhal R.. (2022). Expanded diversity and host range of Bovine hepacivirus-genomic and serological evidence in domestic and wild ruminant species. Viruses 14, 1457. doi: 10.3390/v14071457, PMID: 35891438 PMC9319978

[B7] Bulankina A. V. Richter R. M. Welsch C. (2022). Regulatory role of phospholipids in Hepatitis C virus replication and protein function. Pathogens 11, 102. doi: 10.3390/pathogens11010102, PMID: 35056049 PMC8779051

[B8] Conow C. Fielder D. Ovadia Y. Libeskind-Hadas R. (2010). Jane: a new tool for the cophylogeny reconstruction problem. Algorithm. Mol. Biol. 5, 16. doi: 10.1186/1748-7188-5-16, PMID: 20181081 PMC2830923

[B9] Corman V. M. Grundhoff A. Baechlein C. Fischer N. Gmyl A. Wollny R.. (2015). Highly divergent hepaciviruses from African cattle. J. Virol. 89, 5876–5882. doi: 10.1128/JVI.00393-15, PMID: 25787289 PMC4442428

[B10] de Martinis C. Cardillo L. Esposito C. Viscardi M. Barca L. Cavallo S.. (2022). First identification of bovine hepacivirus in wild boars. Sci. Rep. 12, 11678. doi: 10.1038/s41598-022-15928-7, PMID: 35804025 PMC9270363

[B11] Enomoto N. Sakuma I. Asahina Y. Kurosaki M. Murakami T. Yamamoto C.. (1995). Comparison of full-length sequences of interferon-sensitive and resistant hepatitis C virus 1b. Sensitivity to interferon is conferred by amino acid substitutions in the NS5A region. J. Clin. Invest. 96, 224–230. doi: 10.1172/JCI118025, PMID: 7542279 PMC185192

[B12] Flanagan M. L. Parrish C. R. Cobey S. Glass G. E. Bush R. M. Leighton T. J. (2012). Anticipating the species jump: surveillance for emerging viral threats. Zoonoses Public Health 59, 155–163. doi: 10.1111/j.1863-2378.2011.01439.x, PMID: 21914152 PMC4948863

[B13] Harvey E. Rose K. Eden J. S. Lo N. Abeyasuriya T. Shi M.. (2019). Extensive diversity of RNA viruses in Australian ticks. J. Virol. 93, e01358–e01318. doi: 10.1128/JVI.01358-18, PMID: 30404810 PMC6340049

[B14] Kapoor A. Simmonds P. Gerold G. Qaisar N. Jain K. Henriquez J. A.. (2011). Characterization of a canine homolog of hepatitis C virus. Proc. Natl. Acad. Sci. U.S.A. 108, 11608–11613. doi: 10.1073/pnas.1101794108, PMID: 21610165 PMC3136326

[B15] Lamsal A. Tryland M. Paulsen K. M. Romano J. S. Nymo I. H. Stiasny K.. (2023). Serological screening for tick-borne encephalitis virus in eight Norwegian herds of semi-domesticated reindeer (*Rangifer tarandus tarandus*). Zoonoses Public Health. 70 (8), 692–698. doi: 10.1111/zph.13060, PMID: 37259822

[B16] Li Z. Lin Z. Ba H. Chen L. Yang Y. Wang K.. (2017). Draft genome of the reindeer (Rangifer tarandus). Gigascience 6, 1–5. doi: 10.1093/gigascience/gix102, PMID: 29099922 PMC5726476

[B17] Li L. L. Liu M. M. Shen S. Zhang Y. J. Xu Y. L. Deng H. Y.. (2019). Detection and characterization of a novel hepacivirus in long-tailed ground squirrels (Spermophilus undulatus) in China. Arch. Virol. 164, 2401–2410. doi: 10.1007/s00705-019-04303-z, PMID: 31243554

[B18] Lu G. Ou J. Zhao J. Li S. (2019). Presence of a novel subtype of Bovine hepacivirus in China and expanded classification of Bovine hepacivirus strains worldwide into 7 subtypes. Viruses 11, 843. doi: 10.3390/v11090843, PMID: 31514278 PMC6784114

[B19] Lu Y. Zeng Y. Luo H. Chen N. Zhao L. Zhang H.. (2025). Rapid development of attenuated IBV vaccine candidates through a versatile backbone applicable to variants. NPJ Vaccines 10, 60. doi: 10.1038/s41541-025-01114-z, PMID: 40155419 PMC11953439

[B20] Ma J. Wei Z. Li L. Wang W. Liu Z. Liu N.. (2025). Detection and characterization of bovine hepacivirus in cattle and sheep from Hulunbuir, northeastern China. Front. Cell. Infect. Microbiol. 15. doi: 10.3389/fcimb.2025.1540849, PMID: 39936164 PMC11811627

[B21] Mackenzie J. S. Jeggo M. (2013). Reservoirs and vectors of emerging viruses. Curr. Opin. Virol. 3, 170–179. doi: 10.1016/j.coviro.2013.02.002, PMID: 23491947 PMC7102734

[B22] Martin D. P. Murrell B. Golden M. Khoosal A. Muhire B. (2015). RDP4: Detection and analysis of recombination patterns in virus genomes. Virus Evol. 1, vev003. doi: 10.1093/ve/vev003, PMID: 27774277 PMC5014473

[B23] Morse S. S. Mazet J. A. Woolhouse M. Parrish C. R. Carroll D. Karesh W. B.. (2012). Prediction and prevention of the next pandemic zoonosis. Lancet 380, 1956–1965. doi: 10.1016/S0140-6736(12)61684-5, PMID: 23200504 PMC3712877

[B24] Parola P. (2004). Tick-borne rickettsial diseases: emerging risks in Europe. Comp. Immunol. Microbiol. Infect. Dis. 27, 297–304. doi: 10.1016/j.cimid.2004.03.006, PMID: 15225980

[B25] Paulsen K. M. das Neves C. G. Granquist E. G. Madslien K. Stuen S. Pedersen B. N.. (2020). Cervids as sentinel-species for tick-borne encephalitis virus in Norway - A serological study. Zoonoses Public Health 67, 342–351. doi: 10.1111/zph.12675, PMID: 31855321

[B26] Porter A. F. Pettersson J. H. Chang W. S. Harvey E. Rose K. Shi M.. (2020). Novel hepaci- and pegi-like viruses in native Australian wildlife and non-human primates. Virus Evol. 6, veaa064. doi: 10.1093/ve/veaa064, PMID: 33240526 PMC7673076

[B27] Pybus O. G. Thézé J. (2016). Hepacivirus cross-species transmission and the origins of the hepatitis C virus. Curr. Opin. Virol. 16, 1–7. doi: 10.1016/j.coviro.2015.10.002, PMID: 26517843

[B28] Sánchez Romano J. Grund L. Obiegala A. Nymo I. H. Ancin-Murguzur F. J. Li H.. (2019). A Multi-pathogen screening of captive reindeer (*Rangifer tarandus*) in Germany based on serological and molecular assays. Front. Vet. Sci. 6. doi: 10.3389/fvets.2019.00461, PMID: 31921918 PMC6933772

[B29] Scheel T. K. Gottwein J. M. Mikkelsen L. S. Jensen T. B. Bukh J. (2011). Recombinant HCV variants with NS5A from genotypes 1–7 have different sensitivities to an NS5A inhibitor but not interferon-α. Gastroenterology 140, 1032–1042. doi: 10.1053/j.gastro.2010.11.036, PMID: 21111742

[B30] Shao J. W. Guo L. Y. Yuan Y. X. Ma J. Chen J. M. Liu Q. (2021). A novel subtype of Bovine hepacivirus identified in ticks reveals the genetic diversity and evolution of Bovine Hepacivirus. Viruses 13, 2206. doi: 10.3390/v13112206, PMID: 34835012 PMC8623979

[B31] Sharp P. M. Hahn B. H. (2011). Origins of HIV and the AIDS pandemic. Cold Spring Harb. Perspect. Med. 1, a006841. doi: 10.1101/cshperspect.a006841, PMID: 22229120 PMC3234451

[B32] Shi M. Lin X. D. Chen X. Tian J. H. Chen L. J. Li K.. (2018). The evolutionary history of vertebrate RNA viruses. Nature 556, 197–202. doi: 10.1038/s41586-018-0012-7, PMID: 29618816

[B33] Shi M. Lin X. D. Vasilakis N. Tian J. H. Li C. X. Chen L. J.. (2016). Divergent viruses discovered in arthropods and vertebrates revise the evolutionary history of the Flaviviridae and related viruses. J. Virol. 90, 659–669. doi: 10.1128/JVI.02036-15, PMID: 26491167 PMC4702705

[B34] Simons J. N. Pilot-Matias T. J. Leary T. P. Dawson G. J. Desai S. M. Schlauder G. G.. (1995). Identification of two flavivirus-like genomes in the GB hepatitis agent. Proc. Natl. Acad. Sci. U.S.A. 92, 3401–3405. doi: 10.1073/pnas.92.8.3401, PMID: 7724574 PMC42174

[B35] Slukinova O. S. Kyuregyan K. K. Karlsen A. A. Potemkin I. A. Kichatova V. S. Semenov S. I.. (2021). Serological evidence of Hepatitis E virus circulation among reindeer and reindeer herders. Vector Borne Zoonotic Dis. (Larchmont N.Y.) 21, 546–551. doi: 10.1089/vbz.2020.2727, PMID: 34010077

[B36] Smith D. B. Becher P. Bukh J. Gould E. A. Meyers G. Monath T.. (2016). Proposed update to the taxonomy of the genera Hepacivirus and Pegivirus within the Flaviviridae family. J. Gen. Virol. 97, 2894–2907. doi: 10.1099/jgv.0.000612, PMID: 27692039 PMC5770844

[B37] Walter S. Rasche A. Moreira-Soto A. Pfaender S. Bletsa M. Corman V. M.. (2017). Differential infection patterns and recent evolutionary origins of equine hepaciviruses in donkeys. J. Virol. 91, e01711–e01716. doi: 10.1128/JVI.01711-16, PMID: 27795428 PMC5165184

[B38] Wang B. Yang X. L. Li W. Zhu Y. Ge X. Y. Zhang L. B.. (2017). Detection and genome characterization of four novel bat hepadnaviruses and a hepevirus in China. Virol. J. 14, 40. doi: 10.1186/s12985-017-0706-8, PMID: 28222808 PMC5320732

[B39] Williams S. H. Levy A. Yates R. A. Somaweera N. Neville P. J. Nicholson J.. (2020). Discovery of Jogalong virus, a novel hepacivirus identified in a *Culex annulirostris* (Skuse) mosquito from the Kimberley region of Western Australia. PLoS One 15, e0227114. doi: 10.1371/journal.pone.0227114, PMID: 31899786 PMC6941808

[B40] Yeşilbağ K. Baechlein C. Kadiroğlu B. Baldan Toker E. Alpay G. Becher P. (2018). Presence of bovine hepacivirus in Turkish cattle. Vet. Microbiol. 225, 1–5. doi: 10.1016/j.vetmic.2018.09.001, PMID: 30322519

[B41] Zhai J. C. Liu W. S. Yin Y. J. Xia Y. L. Li H. P. (2017). Analysis on genetic diversity of reindeer (*Rangifer tarandus*) in the Greater Khingan Mountains Using Microsatellite Markers. Zool. Stud. 56, e11. doi: 10.6620/ZS.2017.56-11, PMID: 31966210 PMC6517736

[B42] Zhang X. L. Yao X. Y. Zhang Y. Q. Lv Z. H. Liu H. Sun J.. (2022). A highly divergent hepacivirus identified in domestic ducks further reveals the genetic diversity of hepaciviruses. Viruses 14, 371. doi: 10.3390/v14020371, PMID: 35215964 PMC8879383

