# Supplementary material for: A novel genotype of Hepacivirus bovis identified in reindeer (Rangifer tarandus) in northeastern China
Source: Front Cell Infect Microbiol. 2025 Sep 8;15:1646191. doi: 10.3389/fcimb.2025.1646191 (PMC12450936; doi:10.3389/fcimb.2025.1646191)
Supplement: Supplementary file 1 [file Table1.docx]

**Supplementary Material**

Table S1 The information of primers in this study

| Primer | Position (bp)* | Sequence (5'→3') | Polarity | Amplicon (bp) |
| --- | --- | --- | --- | --- |
| Detection |  |  |  |  |
| F | 85 | CACAAACCCCACGAGTTGAGT | + | 443 |
| R1 | 528 | ACCATCTATSAGACGGCCCAA | - |  |
| R2 | 620 | CTAACCRCCCGACCTAYGAGC | - |  |
| Race |  |  |  |  |
| 5’-GSP | 236 | GATTACGCCAAGCTTAGGACCCTATCAGGCTGTGT |  |  |
| 5’-NGSP | 321 | GATTACGCCAAGCTTTGGACCTGGATCTAGATCT |  |  |
| 3’-GSP 1 | 5784 | GATTACGCCAAGCTTATCGCCAAGCGTGCTGTCG |  |  |
| 3’-NGSP 1 | 5970 | GATTACGCCAAGCTTGCTGGTACAGTCCCTATCA |  |  |
| 3’-GSP 2 | 6998 | GATTACGCCAAGCTTCCATATGGCGGAACGATC |  |  |
| 3’-NGSP 2 | 7029 | GATTACGCCAAGCTTGCGGCGATGACCCATGGC |  |  |
| 3’-GSP 3 | 7772 | GATTACGCCAAGCTTGACTCGCAATTGTCGGGCCT |  |  |
| 3’-NGSP 3 | 8133 | GATTACGCCAAGCTTTCCGCTGAAGGCGATCGG |  |  |
| Genome amplification |  |  |  |  |
| 1 F | 1 | CAACACTCCAGGCCATTGGGT | + | 1050 |
| 1 R1 | 1121 | GCCAGTTAAAGAACCCAATGACT | - |  |
| 1 R2 | 1051 | ATTGAGCTTAATCGACCTAGGCA | - |  |
| 2 F | 899 | CATTAGCCAGCCTAAGAATGC | + | 1150 |
| 2 R1 | 2070 | AAAACAAGGCCAATGAACGC | - |  |
| 2 R2 | 2049 | CGTCCGTGACTTAAACAGACC | - |  |
| 3 F | 1924 | CTGATTTGGCTACTGGACTCA | + | 1003 |
| 3 R1 | 3038 | CGCAAGAGAACCCCATATACCTG | - |  |
| 3 R2 | 2927 | CTACTGGCGACCTTACGGACA | - |  |
| 4 F | 2745 | GTTTTACACGGCGACCCCTA | + | 1006 |
| 4 R1 | 3820 | TTGTCGCTAACACAGAGCCTA | - |  |
| 4 R2 | 3751 | CACGTCAGCTTGGATAGACC | - |  |
| 5 F | 3677 | AGCTGAGATGACCTTAAACACCG | + | 1105 |
| 5 R1 | 4821 | GCCAAGCCCAAAGCAAACAC | - |  |
| 5 R2 | 4782 | TCACAGCACCCCAAACCTC | - |  |
| 6 F | 4138 | TCGCTACAGATGCTTTGATGACT | + | 1019 |
| 6 R1 | 5267 | GTAGCACCAGCAATGAACACC | - |  |
| 6 R2 | 5157 | CCGCACCACTAAAGCTAAGCAA | - |  |
| 7 F | 5074 | CGGCCAATCTCATCACTATTGCT | + | 1162 |
| 7 R1 | 6124 | GAATCACCCTTGGCACGGTCA | - |  |
| 7 R2 | 6236 | TCCCTACCATTGCCAGTGTCC | - |  |
| 8 F | 5970 | GCTGGTACAGTCCCTATCAACA | + | 1005 |
| 8 R1 | 7047 | AGACCAAATTTCGCTGAACCAA | - |  |
| 8 R2 | 6975 | ACATAAGGTATGCCGTCCCA | - |  |
| 9 F | 6870 | GCATCAGAACATAGCTGCACAAC | + | 1088 |
| 9 R1 | 8027 | AACTACAGCTAGTAACGTCCTCC | - |  |
| 9 R2 | 7958 | ATTGCTGAAGCGAAGACACCC | - |  |
| 10 F1 | 7700 | GGCCAGGATCTACAATCTGC | + | 1082 |
| 10 F2 | 7804 | CCACGTCGTCATCCAACACC | + |  |
| 10 R | 8886 | GCGCTGACTAACACCCAAA | - |  |

*Primer positions are referenced to the complete sequences obtained by metagenomic sequencing in this study.

**Table S2.** Reference sequences used in the present study

| Accession number | Strain | Host | Country | Species |
| --- | --- | --- | --- | --- |
| KP265950 | GHC100 | *Bos taurus* | Ghana | *Hepacivirus bovis* |
| MG257793 | BovHepV/GD/01 | *Bos taurus* | China | *Hepacivirus bovis* |
| KP265946 | GHC52 | *Bos taurus* | Ghana | *Hepacivirus bovis* |
| OU592967 | — | *Bos taurus* | — | *Hepacivirus bovis* |
| MH027948 | BH181/16-20 | *Bos taurus* | — | *Hepacivirus bovis* |
| ON402464 | BovHepV_Bulgaria_19 | *Bos taurus* | Bulgaria | *Hepacivirus bovis* |
| MZ221927 | GDZJ | Tick | China | *Hepacivirus bovis* |
| MG781018 | BR_MA236B017 | *Bos taurus* | Brazil | *Hepacivirus bovis* |
| KP641125 | BovHepV_379/Ger/2014 | *Bos taurus* | Germany | *Hepacivirus bovis* |
| MW830376 | CQ/166 | *Bos taurus* | China | *Hepacivirus bovis* |
| OP716809 | HLJ-72 | Tick | China | *Hepacivirus bovis* |
| ON402465 | BovHepV_Bulgaria_9 | *Bos taurus* | Bulgaria | *Hepacivirus bovis* |
| MN691105 | IME_BovHep_01 | *Bos taurus* | China | *Hepacivirus bovis* |
| KP641123 | BovHepV_B1/Ger/2013 | *Bos taurus* | Germany | *Hepacivirus bovis* |
| MH027953 | BH204/16-6 | *Bos taurus* | — | *Hepacivirus bovis* |
| MN266283 | BovHepV/JS/02 | *Bos taurus* | China | *Hepacivirus bovis* |
| MG781019 | BR_RN034B019 | *Bos taurus* | Brazil | *Hepacivirus bovis* |
| MZ540979 | GDZJ02-2 | Tick | China | *Hepacivirus bovis* |
| KP265947 | GHC55 | *Bos taurus* | Ghana | *Hepacivirus bovis* |
| MH027948 | BH181/16-20, | — | — | *Hepacivirus bovis* |
| MG257794 | BovHepV/GD/02 | *Bos taurus* | China | *Hepacivirus bovis* |
| KP265943 | GHC25 | *Bos taurus* | Ghana | *Hepacivirus bovis* |
| MK695669 | BRBovHep_RS963 | *Bos taurus* | Brazil | *Hepacivirus bovis* |
| OQ164634 | GH01 | *Rangifer tarandus* | China | *Hepacivirus bovis* |
| OQ164635 | GH02 | *Rangifer tarandus* | China | *Hepacivirus bovis* |
| KC796077 | PDB-112 | *Hipposideros vittatus* | Kenya | *Hepacivirus vittatae* |
| KC551800 | GHV-1 BWC08 | Black-and-white colobus | Uganda | *Hepacivirus colobi* |
| U22304 | — | — | — | *Hepacivirus platyrrhini* |
| KJ950939 | NrHV-2/NYC-E43 | *Rattus norvegicus* | USA | *Hepacivirus norvegici* |
| MG211815 | RHV-GS2015 | *Citellus dauricus Brandt* | China | — |
| KJ950938 | NrHV-1/NYC-C12 | *Rattus norvegicus* | USA | *Hepacivirus ratti* |
| KC815310 | RHV-339 | *Peromyscus maniculatus* | USA | *Hepacivirus peromysci* |
| KC411784 | Hepacivirus/NLR07-oct70/NEL/2007 | *Myodes glareolu* | Netherlands | *Hepacivirus myodae* |
| KC411806 | Hepacivirus/SAR-3/RSA/2008 | *Rhabdomys pumilio* | South Africa | *Hepacivirus rhabdomysis* |
| KC411777 | Hepacivirus/RMU10-3382/GER/2010 | *Myodes glareolu* | Germany | *Hepacivirus glareoli* |
| KC796074 | PDB-829 | *Hipposideros vittatus* | Kenya | *Hepacivirus macronycteridis* |
| KC796078 | PDB-491.1 | *Otomops martiensseni* | Kenya | *Hepacivirus otomopis* |
| JF744991 | AAK-2011 | Canine | USA | — |
| KP325401 | NZP1 | Horse | — | *Hepacivirus equi* |
| NC009826 | EUH1480 | *Homo sapiens* | UK | *Hepacivirus hominis* |
| NC009827 | Th580 | *Homo sapiens* | — | *Hepacivirus hominis* |
| OM203121 | GDZQ-15 | Goose | China | — |
